# Supplementary material for: Improvement of Cognitive Function by Fermented Panax ginseng C.A. Meyer Berries Extracts in an AF64A-Induced Memory Deficit Model
Source: Nutrients. 2023 Jul 30;15(15):3389. doi: 10.3390/nu15153389 (PMC10421307; doi:10.3390/nu15153389)
Supplement: Supplementary file 1 [file nutrients-15-03389-s001.zip › nutrients-2435607-supplementary.pdf]

Supplementary Table S1. Primer sequences used for PCR analysis in this study

A. Primer Sequences used for human NSCs			
Gene	Primer sequences		Accession No.
	Forward	Reverse	
CHT1	TGCAGTATCTCTGCCCTGTG	TGTGATTCGCATAACCCAAA	NM_021815
ChAT	TCATTAATTTCCGCCGTCTC	GAGTCCCGGTTGGTGGAGT	NM_020986
VACHT	GTGCGCCATGTCTCAGTCTA	CATAGAGCAGGTTGGCCAGT	NM_003055
M1ACHR	AGCTCCCCAAATACAGTC	TTGCAGAGTGCGTAGCAG	NM_000738
nAChRα5	TTTCTTCACACGCTTCCCAAA	TCACGGACATCATTTTCCTTCA	NM_000745
nAChRβ2	GCCTGCGCCTGCGGCGACGCC	CCTCACTCACGCTCTGGTCAT	NG_008027
BDNF	GGATGAGGACCAGAAAGT	AGCAGAAAGAGAAGAGGAG	NM_170734
NGF	CCATCCCATCTTCCACAG	CTCTCCCAACACCATCAC	NM_002506
GAPDH	AAGAAGGTGGTGAAGCAG	GTCAAAGGTGGAGGAGTG	NM_001289746
B. Primer sequences used for mice brain			
CHT1	TTCCAGATTCAGGCAGTAGACG	GGGAGGGAAACTCCTATCTTGT	NM_022025
ChAT	TGGGTCTCTGAATACTGGCTGA	GGGCTAGAGTTGACTGGCAGG	NM_009891
VACHT	CCCTTTTGATGGCTGTGA	GGGCTAGGGTACTCATTAGA	NM_021712
M1ACHR	TGACAGGCAACCTGCTGGTGCT	AATCATCAGAGCTGCCCTGCGG	NM_007698
nAChRα5	GAGCAAGGGGAACCGGAC	TCTTACGAACCCACGGCG	NM_176844
nAChRβ2	CTCCTTTGGCTGTGTTCAGG	AGGAGACTTCGTACATGCCG	NM_009602
BDNF	TACTTCGGTTGCATGAAGGCG	GTCAGACCTCTCGAACCTGCC	NM_007540
NGF	TGATCGGCGTACAGGCAGA	GAGGGCTGTGTCAAGGGAAT	NM_013609
GAPDH	CGTGCCGCCTGGAGAAACC	TGGAAGAGTGGGAGTTGCTGTTG	NM_008084

Supplementary Table S2. List of antibodies used in the current study

Epitope	Company	Cat. No.	Dilution	2° Ab (IgG)
CHT (42 kDa)	Abcam	ab154186	1:1000	Rb
ChAT (82 kDa)	Abcam	ab178850	1:1000	Rb
VAChT (56 kDa)	Synaptic system	#139103	1:500	Rb
BDNF (14 kDa)	Abcam	ab226843	1:1000	Rb
NGF (13 kDa)	Abcam	ab6199	1:1000	Rb
GFAP (55 kDa)	Abcam	Ab7260	1:10000	Rb
Abeta (4 kDa)	MyBiosource	MBS632680	1:500	Ms
Actin (43 kDa)	Cell Signal	#5125	1:1000	Rb

Supplementary Table S3. Contents of ginsenoside in pre- and post-fermented GBE (mg/g)

Peak No.	Ginsenoside	Pre	Post	Changes (average, n=3)
1	Rg1	5.79±0.11	-	-5.79
2	Re	84.72±0.28	1.31±0.09	-83.40
3	Rb1	11.34±0.23	-	-11.34
4	Rc	12.95±0.12	5.21±0.19	-7.74
5	Rb2	20.2±0.17	2.39±0.1	-17.81
6	F1	1.17±0.05	-	-1.17
7	Rd	42.45±0.06	14.21±0.2	-28.24
8	F4	4.32±0.1	58.47±0.35	+54.15
9	F2	0.61±0.03	5.36±0.12	+4.75
10	Rg3(S)	1.22±0.06	26.34±0.1	+25.12
11	Rg3(R)	0.18±0.01	17.79±0.07	+17.61
12	Rk1	1.78±0.06	10.12±0.08	+8.34
13	Rg5	0.64±0.03	12.33±0.04	+11.69
14	Rh2	-	0.23±0.02	+0.23
	Total	187.36±1.15	153.75±1.23	-33.61

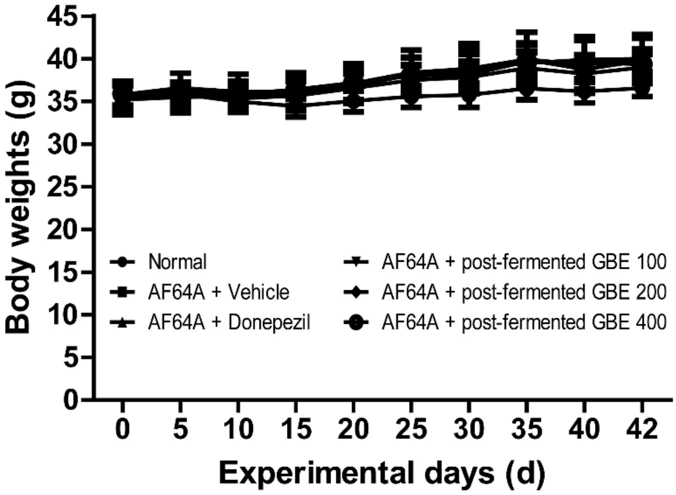

Supplementary Figure S1. The body weights during experimental period. The animals were orally administrated with donepezil (2 mg/kg) and GBE (100, 200, and 400 mg/kg) during 6 weeks. n=7 in each group. Data are presented as mean ± SD.
